# Editorial

**Published:** 1844-01-27

**Authors:** 


### THE MEDICAL EXAMINER.

### PHILADELPHIA, JANUARY 27, 1844.

### MEDICAL SOCIETY.

At a late meeting of the Philadelphia Medical Society, Professors Chomel and Andral, of Paris, and Dr. Fleetwood Churchill, of Dublin, were unanimously elected honorary members.

### CLINICAL INSTRUCTION.

The means of Clinical Instruction afforded by Philadelphia are yearly becoming more developed. With the exception of Paris and London, in no city perhaps at this moment is greater attention paid to Clinical medicine, or better opportunities presented to students for acquiring a practical knowledge of the medical profession. We rejoice at this. As "the metropolis of medical science" on the American Continent, it is incumbent on her to be foremost in the march of improvement. In addition to the Hospitals and Dispensaries, for several years past a Clinic has been regularly held at Jefferson Medical College, at which more than a thousand cases have been prescribed for annually; and, recently, a Surgical Clinic has been established by the University of Pennsylvania, where we understand several interesting operations have been performed, of which we shall be glad to receive *reports.*

### MEDICAL STUDENTS' TEMPERANCE SOCIETY.

The improvement in education and general character of the Medical Students at the Colleges in Philadelphia, within the last few years, is the common subject of remark with all who have had the opportunity of judging. There are at this time between seven and eight hundred of these young gentlemen in this city,—collected from nearly all parts of the continent and the adjacent islands, surrounded by the temptations of a large city, and without the restraining presence of parents and relations,—as quietly and diligently engaged in the pursuit of knowledge as any gray-headed philosophers that ever congregated together. Months have now elapsed since many of them arrived here, and yet, up to this hour, no outbreak, no unbecoming exposure of themselves, has occurred in any instance, so far as we have been able to learn. They afford an example, indeed, to the young men of other professions in the place, which it would be profitable for them to follow. Among other evidences of their self denial and rigid determination to keep out of the way of temptation, is their voluntary association as members of a temperance society, on the principle of total abstinence. Early in the session of last year such a society was formed among them, and embraced a considerable number;—the present winter a similar movement was made early in the session; two public meetings have been held, at which nearly all the students in the city were present, and a very large number signed the pledge.

### PREGNANCY, TWO YEARS AFTER CESSATION OF THE MENSES.

At a recent meeting of the "Societé Medicale du Temple," M. Legros narrated the following case:—A married woman, mother of several children, ceased to menstruate at the age of forty-one. Two years afterwards her general health having become disordered, she consulted M. Legros. She was then thin sallow, and presented other symptoms which seemed to indicate a cancerous cachexia. She herself stated that she thought she was pregnant. M. Legros, considering that the menstrual function had been entirely absent for two years, and that its cessation had been accompanied by the symptoms which usually attend its final disappearance, thought he had, in all probability, a cancerous affection of the uterus to deal with, and prescribed an appropriate treatment. He does not appear to have examined the state of the internal organs of generation per vaginam, a most egregious error in such a case, as the neglect of this means of diagnosis, rendered it next to impossible to arrive at a correct opinion of the state of the patient. A few months afterwards the woman was delivered of a full-grown child. She then confessed that on thinking herself safe, she had abandoned herself to a young man. M. Legros suggests that the excitement which attended this new *liaison* may have revived the functional vitality of the uterus, and this seems indeed to be the most rational view of the case.—*Lon. Med. Gaz., from Gaz. Med.*

